# (*R*,*R*)-Disynephrine ether bis­(hydrogen sulfate)

**DOI:** 10.1107/S1600536809025288

**Published:** 2009-07-04

**Authors:** William Arbuckle, Alan R. Kennedy, Catriona A. Morrison

**Affiliations:** a Schering-Plough Research Institute, Newhouse, Motherwell ML1 5SH, Scotland; b Department of Pure & Applied Chemistry, University of Strathclyde, 295 Cathedral Street, Glasgow G1 1XL, Scotland

## Abstract

The asymmetric unit of the title compound [systematic name: (*R*,*R*)-2,4-bis­(4-hydroxy­phen­yl)-*N*,*N*′-dimethyl-3-oxapentane-1,5-diammonium bis­(hydrogen sulfate)], C_18_H_26_N_2_O_3_^2+^·2HSO_4_^−^, contains one half-cation and one hydrogen sulfate anion. The cation has crystallographically imposed twofold symmetry with the rotation axis passing through the central ether O atom. Hydrogen bonds between the hydr­oxy group and amine H atoms of the cation to two hydrogen sulfate anions link the three ions in a ring motif. A three-dimensional network is accomplished by additional O—H⋯O hydrogen bonds between the anions and by N—H⋯O hydrogen bonds between the cations. Disorder with equally occupied sites affects the H-atom position in the anion.

## Related literature

For the preparation and structure of the equivalent bromide salt, see: Mukhopadhyay & Dattagupta (1984 ▶, 1988 ▶). For recent examples of synephrine use, see: Blanck *et al.* (2007 ▶): Haller *et al.* (2008 ▶). For general background, see: Bruice (2007 ▶); Jacques *et al.* (1981 ▶).
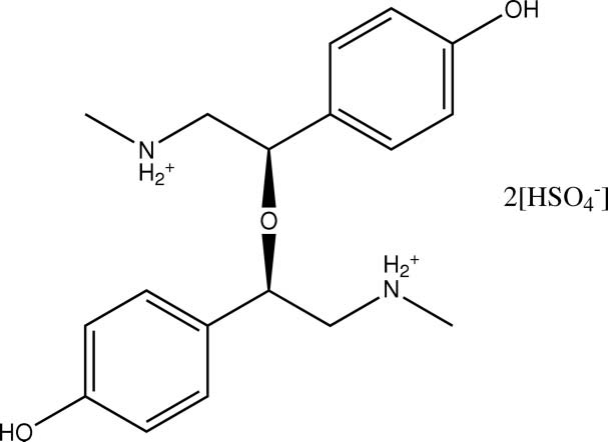


## Experimental

### 

#### Crystal data

C_18_H_26_N_2_O_3_^2+^·2HSO_4_^−^
*M* *_r_* = 512.54
Monoclinic, 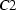

*a* = 13.7204 (9) Å
*b* = 11.5853 (5) Å
*c* = 7.6579 (5) Å
β = 116.413 (8)°
*V* = 1090.19 (13) Å^3^
*Z* = 2
Mo *K*α radiation
μ = 0.31 mm^−1^
*T* = 123 K
0.23 × 0.15 × 0.11 mm

#### Data collection

Oxford Diffraction Gemini S CCD diffractometer
Absorption correction: multi-scan (ABSPACK; Oxford Diffraction, 2007 ▶) *T*_min_ = 0.974, *T*_max_ = 1.000 (expected range = 0.942–0.967)
5888 measured reflections
2401 independent reflections
2034 reflections with *I* > 2σ(*I*)
*R*_int_ = 0.027

#### Refinement

*R*[*F* ^2^ > 2σ(*F* ^2^)] = 0.043
*wR*(*F* ^2^) = 0.111
*S* = 1.03
2401 reflections
161 parameters
1 restraint
H atoms treated by a mixture of independent and constrained refinement
Δρ_max_ = 0.57 e Å^−3^
Δρ_min_ = −0.43 e Å^−3^
Absolute structure: Flack (1983 ▶), 1032 Friedel pairs
Flack parameter: 0.08 (11)

### 

Data collection: *CrysAlis CCD* (Oxford Diffraction, 2007 ▶); cell refinement: *CrysAlis CCD*; data reduction: *CrysAlis RED* (Oxford Diffraction, 2007 ▶); program(s) used to solve structure: *SHELXS97* (Sheldrick, 2008 ▶); program(s) used to refine structure: *SHELXL97* (Sheldrick, 2008 ▶); molecular graphics: *ORTEP-3* (Farrugia, 1997 ▶); software used to prepare material for publication: *SHELXL97*.

## Supplementary Material

Crystal structure: contains datablocks global, I. DOI: 10.1107/S1600536809025288/wm2243sup1.cif

Structure factors: contains datablocks I. DOI: 10.1107/S1600536809025288/wm2243Isup2.hkl

Additional supplementary materials:  crystallographic information; 3D view; checkCIF report

## Figures and Tables

**Table 1:** Hydrogen-bond geometry (Å, °)

*D*—H⋯*A*	*D*—H	H⋯*A*	*D*⋯*A*	*D*—H⋯*A*
O1—H1⋯O3^i^	0.84	1.95	2.739 (3)	155
N1—H1*N*⋯O4^ii^	0.83 (3)	1.93 (3)	2.700 (4)	153 (3)
N1—H2*N*⋯O1^iii^	0.82 (3)	2.27 (3)	2.999 (3)	149 (3)
O5—H1*S*⋯O5^iv^	0.91	1.65	2.502 (6)	155
O6—H2*S*⋯O6^i^	1.05	1.60	2.493 (5)	139

Symmetry codes: (i) 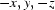; (ii) 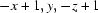; (iii) 
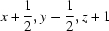
; (iv) 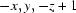.

**Fig. 1.**
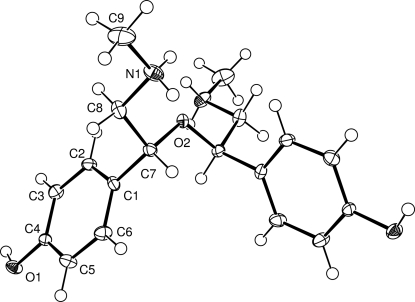
The molecular structure and atomic labelling of the cation, showing 50% probability displacement ellipsoids. H atoms are shown as spheres of arbitrary radius.

**Fig. 2.**
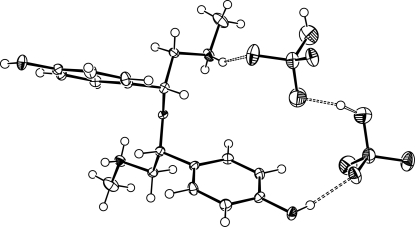
The hydrogen-bonded ring motif between one cation and two anions in the structure of [C18H26N2O3][SO4H]2.

**Fig. 3.**
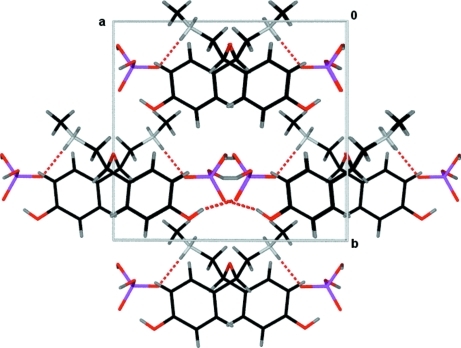
Packed structure viewed down the c-axis. S-atoms are pink and O-atoms are red. Hydrogen-bonding is shown as dashed lines.

## supplementary crystallographic information

### Comment

Synephrine (systematic name 2-hydroxy-2-(4-hydroxyphenyl)-*N*-methylethanamine) is a member of the phenylethylamine drug family and it is often found in products marketed as "traditional medicines" or "weight-loss" pills (Blanck *et al.*, 2007; Haller *et al.*, 2008). An attempt to prepare the sulfate salt of a racemic sample gave instead only the title compound, di-synephrine ether di-hydrogen sulfate, presumably *via* a variation of the well known S_N_^2^ condensation reaction (Mukhopadhyay & Dattagupta, 1988; Bruice, 2007).

The salt crystallizes in space group *C*2, with half a cation and one hydrogen sulfate anion in the asymmetric unit. The twofold rotation axis passes through O2, the etheric O atom (Fig. 1). The (*R*,*R*) conformation was assigned after refinement of the Flack parameter (0.08 (11)). The bulk sample is presumably thus a conglomerate (Jacques *et al.*, 1981). Similar symmetry is seen in the molecular structure of the analogous bromide salt (Mukhopadhyay & Dattagupta, 1988). Despite the gross structural similarities imposed by the identical symmetries, the two salts do have somewhat different conformations. This is best illustrated by the C8—C7—C7*—C8* torsion angle (-28.7 (3) ° here and -11.3 ° in the Br salt). Disorder effects the H atom position in the [HSO_4_] anion, with equally occupied proton sites (50:50) associated with both O5 and O6.

Hydrogen bonds (Table 1) from the cation's hydroxy and amine H atoms to two [HSO_4_] anions link the three ions (one cation and two anions) in a ring motif (Fig. 2). Further anion to anion hydrogen bonded interactions give columns of [HSO_4_] lying along the *c* direction and complete a hydrogen bonded network in the *ac* plane (Fig. 3). A final, weaker cation to cation interaction between the NH_2_ and –OH links these planes in the *b* direction.

### Experimental

The title compound was obtained on treating an aqueous solution of (+/-)synephrine with dilute sulfuric acid. Single-crystals were obtained by allowing the solvent of the reaction mixture to evaporate at 295 K. ^1^H NMR (DMSO-d6) 9.67 (2*H*, s br, OH); 8.40 (4*H*, s br, NH_2_); 7.07 (4*H*, d, *sp*^2^ CH); 6.80 (4*H*, d, *sp*^2^ CH); 4.20 (2*H*, dd, OCH); 3.32 (2*H*, m, CH_2_); 2.99 (2*H*, m, CH_2_); 2.57 (6*H*, t, Me).

### Refinement

Amine H atomes were found by difference synthesis and refined isotropically. All other H atoms of the cation were positioned geometrically at distances of 0.95, 1.00, 0.99, 0.98 and 0.84 Å from the parent atoms for CH(ar), CH(*sp*^3^), CH_2_, CH_3_ and ROH groups respectively. For these groups a riding model was used with *U*_iso_(H) values constrained to be 1.2 times *U*_eq_ of the parent atom for CH and CH_2_ groups and 1.5 times *U*_eq_ of the parent atom for OH and CH_3_ groups. The anion's proton was found by difference synthesis to be disordered over two equally occupied sites. These were placed as found and constrained to ride on the parent O atoms with *U*_iso_(H) equal to 1.5 times *U*_eq_ of the parent atom.

### Crystal data

**Table:**

C_18_H_26_N_2_O_3_^2+^·2HSO_4_^−^	*F*(000) = 540
*M**_r_* = 512.54	*D*_x_ = 1.561 Mg m^−^^3^
Monoclinic, *C*2	Mo *K*α radiation, λ = 0.71073 Å
Hall symbol: C 2y	Cell parameters from 3122 reflections
*a* = 13.7204 (9) Å	θ = 2.4–29.1°
*b* = 11.5853 (5) Å	µ = 0.31 mm^−^^1^
*c* = 7.6579 (5) Å	*T* = 123 K
β = 116.413 (8)°	Prism, colourless
*V* = 1090.19 (13) Å^3^	0.23 × 0.15 × 0.11 mm
*Z* = 2	

### Data collection

**Table:**

Oxford Diffraction Gemini S CCD diffractometer	2401 independent reflections
Radiation source: fine-focus sealed tube	2034 reflections with *I* > 2σ(*I*)
graphite	*R*_int_ = 0.027
ω scans	θ_max_ = 28.0°, θ_min_ = 2.4°
Absorption correction: multi-scan (ABSPACK; Oxford Diffraction, 2007)	*h* = −17→16
*T*_min_ = 0.974, *T*_max_ = 1.000	*k* = −14→14
5888 measured reflections	*l* = −10→10

### Refinement

**Table:**

Refinement on *F*^2^	Secondary atom site location: difference Fourier map
Least-squares matrix: full	Hydrogen site location: inferred from neighbouring sites
*R*[*F*^2^ > 2σ(*F*^2^)] = 0.043	H atoms treated by a mixture of independent and constrained refinement
*wR*(*F*^2^) = 0.111	*w* = 1/[σ^2^(*F*_o_^2^) + (0.071*P*)^2^] where *P* = (*F*_o_^2^ + 2*F*_c_^2^)/3
*S* = 1.03	(Δ/σ)_max_ < 0.001
2401 reflections	Δρ_max_ = 0.57 e Å^−^^3^
161 parameters	Δρ_min_ = −0.43 e Å^−^^3^
1 restraint	Absolute structure: Flack (1983), 1032 Friedel pairs
Primary atom site location: structure-invariant direct methods	Flack parameter: 0.08 (11)

### Special details

**Table:**

Geometry. All e.s.d.'s (except the e.s.d. in the dihedral angle between two l.s. planes) are estimated using the full covariance matrix. The cell e.s.d.'s are taken into account individually in the estimation of e.s.d.'s in distances, angles and torsion angles; correlations between e.s.d.'s in cell parameters are only used when they are defined by crystal symmetry. An approximate (isotropic) treatment of cell e.s.d.'s is used for estimating e.s.d.'s involving l.s. planes.
Refinement. Refinement of *F*^2^ against ALL reflections. The weighted *R*-factor *wR* and goodness of fit *S* are based on *F*^2^, conventional *R*-factors *R* are based on *F*, with *F* set to zero for negative *F*^2^. The threshold expression of *F*^2^ > σ(*F*^2^) is used only for calculating *R*-factors(gt) *etc*. and is not relevant to the choice of reflections for refinement. *R*-factors based on *F*^2^ are statistically about twice as large as those based on *F*, and *R*- factors based on ALL data will be even larger.

### Fractional atomic coordinates and isotropic or equivalent isotropic displacement parameters (Å^2^)

**Table:**

	*x*	*y*	*z*	*U*_iso_*/*U*_eq_	Occ. (<1)
S1	0.08291 (6)	0.20896 (7)	0.33257 (10)	0.0289 (2)	
O1	0.17446 (14)	0.40319 (17)	−0.2357 (3)	0.0181 (4)	
H1	0.1185	0.3616	−0.2796	0.027*	
O2	0.5000	0.1062 (2)	0.5000	0.0128 (5)	
O3	0.02688 (19)	0.31541 (19)	0.3192 (4)	0.0304 (6)	
O4	0.19286 (16)	0.2108 (3)	0.4864 (3)	0.0395 (6)	
O5	0.0193 (2)	0.1104 (2)	0.3550 (4)	0.0456 (7)	
H1S	0.0204	0.1255	0.4724	0.068*	0.50
O6	0.0896 (2)	0.1834 (3)	0.1477 (4)	0.0519 (8)	
H2S	0.0295	0.2160	0.0149	0.078*	0.50
N1	0.6712 (2)	0.0279 (2)	0.4186 (4)	0.0191 (5)	
C1	0.4315 (2)	0.2368 (2)	0.2158 (3)	0.0135 (6)	
C2	0.3329 (2)	0.1795 (2)	0.1126 (4)	0.0156 (6)	
H2	0.3247	0.1025	0.1470	0.019*	
C3	0.2473 (2)	0.2329 (2)	−0.0382 (4)	0.0166 (6)	
H3	0.1807	0.1927	−0.1078	0.020*	
C4	0.2581 (2)	0.3458 (2)	−0.0891 (4)	0.0139 (5)	
C5	0.3554 (2)	0.4041 (3)	0.0124 (4)	0.0182 (6)	
H5	0.3633	0.4812	−0.0217	0.022*	
C6	0.4414 (2)	0.3491 (2)	0.1646 (4)	0.0173 (6)	
H6	0.5079	0.3893	0.2346	0.021*	
C7	0.5277 (2)	0.1742 (2)	0.3714 (4)	0.0136 (5)	
H7	0.5851	0.2315	0.4495	0.016*	
C8	0.5734 (2)	0.0900 (3)	0.2748 (4)	0.0172 (6)	
H8A	0.5165	0.0329	0.1989	0.021*	
H8B	0.5928	0.1326	0.1827	0.021*	
C9	0.7267 (3)	−0.0381 (3)	0.3240 (5)	0.0302 (7)	
H9A	0.7837	−0.0864	0.4212	0.045*	
H9B	0.7595	0.0155	0.2662	0.045*	
H9C	0.6738	−0.0873	0.2216	0.045*	
H1N	0.711 (2)	0.082 (3)	0.482 (4)	0.013 (8)*	
H2N	0.648 (3)	−0.011 (3)	0.480 (4)	0.012 (8)*	

### Atomic displacement parameters (Å^2^)

**Table:**

	*U*^11^	*U*^22^	*U*^33^	*U*^12^	*U*^13^	*U*^23^
S1	0.0228 (4)	0.0289 (4)	0.0312 (4)	0.0004 (4)	0.0086 (3)	−0.0031 (4)
O1	0.0142 (9)	0.0149 (9)	0.0191 (10)	0.0002 (8)	0.0019 (8)	0.0039 (8)
O2	0.0139 (12)	0.0143 (13)	0.0101 (11)	0.000	0.0053 (10)	0.000
O3	0.0196 (11)	0.0272 (12)	0.0420 (13)	0.0024 (9)	0.0117 (10)	−0.0016 (10)
O4	0.0275 (11)	0.0363 (12)	0.0388 (12)	0.0082 (14)	0.0005 (9)	0.0003 (14)
O5	0.0485 (16)	0.0330 (14)	0.0552 (17)	−0.0104 (13)	0.0231 (14)	−0.0060 (13)
O6	0.0427 (15)	0.072 (2)	0.0398 (14)	0.0081 (15)	0.0172 (13)	−0.0090 (13)
N1	0.0160 (12)	0.0187 (13)	0.0244 (12)	0.0036 (10)	0.0106 (11)	0.0065 (11)
C1	0.0139 (13)	0.0157 (15)	0.0108 (11)	0.0032 (9)	0.0054 (10)	0.0027 (9)
C2	0.0190 (13)	0.0106 (13)	0.0178 (12)	−0.0004 (10)	0.0088 (11)	0.0009 (9)
C3	0.0134 (13)	0.0172 (16)	0.0178 (12)	−0.0026 (10)	0.0057 (10)	−0.0019 (10)
C4	0.0164 (13)	0.0118 (12)	0.0125 (12)	0.0040 (10)	0.0055 (11)	0.0010 (10)
C5	0.0184 (14)	0.0138 (12)	0.0217 (14)	0.0003 (12)	0.0082 (12)	0.0044 (11)
C6	0.0135 (13)	0.0140 (13)	0.0224 (13)	−0.0030 (11)	0.0063 (11)	−0.0010 (11)
C7	0.0143 (12)	0.0120 (12)	0.0133 (12)	−0.0016 (9)	0.0053 (10)	0.0011 (9)
C8	0.0145 (13)	0.0239 (15)	0.0148 (12)	0.0049 (11)	0.0079 (10)	0.0031 (11)
C9	0.0292 (18)	0.0236 (17)	0.047 (2)	0.0087 (13)	0.0251 (16)	0.0051 (14)

### Geometric parameters (Å, °)

**Table:**

S1—O3	1.433 (2)	C1—C7	1.513 (3)
S1—O4	1.443 (2)	C2—C3	1.375 (4)
S1—O6	1.488 (3)	C2—H2	0.9500
S1—O5	1.492 (3)	C3—C4	1.392 (4)
O1—C4	1.368 (3)	C3—H3	0.9500
O1—H1	0.8400	C4—C5	1.386 (4)
O2—C7	1.438 (3)	C5—C6	1.390 (4)
O2—C7^i^	1.438 (3)	C5—H5	0.9500
O5—H1S	0.9095	C6—H6	0.9500
O6—H2S	1.0540	C7—C8	1.517 (4)
N1—C9	1.477 (4)	C7—H7	1.0000
N1—C8	1.489 (3)	C8—H8A	0.9900
N1—H1N	0.83 (3)	C8—H8B	0.9900
N1—H2N	0.82 (3)	C9—H9A	0.9800
C1—C6	1.383 (4)	C9—H9B	0.9800
C1—C2	1.395 (4)	C9—H9C	0.9800
			
O3—S1—O4	112.25 (15)	O1—C4—C3	122.1 (2)
O3—S1—O6	111.20 (16)	C5—C4—C3	119.8 (2)
O4—S1—O6	107.16 (15)	C4—C5—C6	119.6 (3)
O3—S1—O5	110.11 (14)	C4—C5—H5	120.2
O4—S1—O5	111.70 (16)	C6—C5—H5	120.2
O6—S1—O5	104.10 (17)	C1—C6—C5	121.0 (2)
C4—O1—H1	109.5	C1—C6—H6	119.5
C7—O2—C7^i^	113.6 (3)	C5—C6—H6	119.5
S1—O5—H1S	102.3	O2—C7—C1	113.45 (19)
S1—O6—H2S	119.6	O2—C7—C8	105.9 (2)
C9—N1—C8	112.3 (2)	C1—C7—C8	109.2 (2)
C9—N1—H1N	109 (2)	O2—C7—H7	109.4
C8—N1—H1N	102 (2)	C1—C7—H7	109.4
C9—N1—H2N	114 (2)	C8—C7—H7	109.4
C8—N1—H2N	104 (2)	N1—C8—C7	112.5 (2)
H1N—N1—H2N	114 (3)	N1—C8—H8A	109.1
C6—C1—C2	118.7 (2)	C7—C8—H8A	109.1
C6—C1—C7	120.9 (2)	N1—C8—H8B	109.1
C2—C1—C7	120.3 (2)	C7—C8—H8B	109.1
C3—C2—C1	120.8 (2)	H8A—C8—H8B	107.8
C3—C2—H2	119.6	N1—C9—H9A	109.5
C1—C2—H2	119.6	N1—C9—H9B	109.5
C2—C3—C4	120.1 (2)	H9A—C9—H9B	109.5
C2—C3—H3	120.0	N1—C9—H9C	109.5
C4—C3—H3	120.0	H9A—C9—H9C	109.5
O1—C4—C5	118.1 (2)	H9B—C9—H9C	109.5
			
C6—C1—C2—C3	0.6 (4)	C7^i^—O2—C7—C1	−70.07 (17)
C7—C1—C2—C3	−175.4 (2)	C7^i^—O2—C7—C8	170.1 (2)
C1—C2—C3—C4	−0.4 (4)	C6—C1—C7—O2	139.0 (2)
C2—C3—C4—O1	−178.7 (2)	C2—C1—C7—O2	−45.1 (3)
C2—C3—C4—C5	0.2 (4)	C6—C1—C7—C8	−103.1 (3)
O1—C4—C5—C6	178.8 (2)	C2—C1—C7—C8	72.8 (3)
C3—C4—C5—C6	−0.1 (4)	C9—N1—C8—C7	−169.9 (2)
C2—C1—C6—C5	−0.5 (4)	O2—C7—C8—N1	−59.6 (3)
C7—C1—C6—C5	175.4 (2)	C1—C7—C8—N1	177.9 (2)
C4—C5—C6—C1	0.3 (4)		

Symmetry codes: (i) −*x*+1, *y*, −*z*+1.

### Hydrogen-bond geometry (Å, °)

**Table:**

*D*—H···*A*	*D*—H	H···*A*	*D*···*A*	*D*—H···*A*
O1—H1···O3^ii^	0.84	1.95	2.739 (3)	155
N1—H1N···O4^i^	0.83 (3)	1.93 (3)	2.700 (4)	153 (3)
N1—H2N···O1^iii^	0.82 (3)	2.27 (3)	2.999 (3)	149 (3)
O5—H1S···O5^iv^	0.91	1.65	2.502 (6)	155
O6—H2S···O6^ii^	1.05	1.60	2.493 (5)	139

Symmetry codes: (ii) −*x*, *y*, −*z*; (i) −*x*+1, *y*, −*z*+1; (iii) *x*+1/2, *y*−1/2, *z*+1; (iv) −*x*, *y*, −*z*+1.
